# Unravelling drivers of the future Mediterranean precipitation paradox during cyclones

**DOI:** 10.21203/rs.3.rs-6211691/v1

**Published:** 2025-03-17

**Authors:** Marco Chericoni, Giorgia Fosser, Alessandro Anav, Emmanouil Flaounas, Marco Gaetani

**Affiliations:** University School for Advanced Studies IUSS; University School for Advanced Studies IUSS; Italian National Agency for New Technologies, Energy and the Environment (ENEA); Hellenic Centre for Marine Research (HCMR); University School for Advanced Studies IUSS

## Abstract

Climate models indicate that the Mediterranean region is projected to experience both general drying and increased cyclone-related precipitation, intensifying flood risks, especially under high-emission scenarios. The use of high-resolution atmosphere-ocean coupled climate models allows to identify the key role of moisture advection and convection processes in cyclone-related precipitation thanks to the more realistic representation of ocean dynamics and air-sea interactions, that low-resolution global models struggle to capture.

## Main text

Recent floods in southern Europe, primarily driven by extratropical cyclones and intensified by climate change, have had severe socioeconomic consequences. Notable episodes include the floods in central Italy in 2023^1^ and in Valencia in 2024^2^, both resulted in large economic losses, societal disruption, and fatalities. Additionally, the Mediterranean basin is increasingly recognized as a climate change hotspot^3,4^. For these reasons, understanding the impact of climate change on the interplay between cyclones and precipitation is more critical than ever.

Cyclogenesis in the Mediterranean region is driven by the intrusion of upper-tropospheric systems, caused by Rossby wave breaking over the Atlantic Ocean^5^. These systems are stronger and more frequent during the extended cold season (October-March, ONDJFM)^6^. Cyclone development then depends on both large-scale baroclinic forcing and deep convection near the cyclone centre, which generates potential vorticity critical for its intensification^7^. Maximum intensity, denoted by the lowest sea level pressure (SLP), tend to occur over maritime areas, in the Gulf of Genoa and in the Adriatic, as well as in the Ionian and Aegean Seas^5^. Global warming is expected to increase atmospheric moisture and potential energy available for convection at the surface, fostering moisture convergence, uplift and thus the precipitable water during intense cyclones^8^. However, changes in baroclinic forcing can either amplify or inhibit these processes, highlighting the complex interplay of factors influencing precipitation response to climate change in the Mediterranean region.

Previous studies based on CMIP5^6,9^ highlight a climate paradox for future Mediterranean precipitation: while mean precipitation is expected to decline, leading to aridification and water scarcity^10^ due to reduced cyclonic activity, cyclone-related precipitation is projected to increase in the north-central Mediterranean but decrease in the southeast. However, there is a pronounced inter-model spread regarding these changes, and the underlying mechanisms are not yet fully understood.

This study aims to bridge these knowledge gaps by investigating how changes in moisture advection and convection processes locally impact cyclone-related precipitation^11^. A better understanding of these processes is essential for improving confidence in climate projections and attributing changes to climate change^12^. Both global (CMIP6, Coupled Model Intercomparison Project Phase 6) and regional (ENEA-REG^13^, Table 1 in online content) climate projections are used to evaluate the changes in Mediterranean cyclones characteristics and associated precipitation under three Shared Socioeconomic Pathways^14^ (SSP5–8.5, SSP2–4.5, and SSP1–2.6). Notably, ENEA-REG is the only available high-resolution atmosphere-ocean coupled regional climate model (AORCM) in the Med-CORDEX initiative^15^ that supports all three SSP scenarios. Mediterranean cyclones are tracked in all simulations within the Med-CORDEX domain (Fig. S1), for the period 1982–2100. The analysis focuses on the most intense cyclones, defined as those with a minimum SLP below 1000 hPa, during the extended cold season, when their influence on precipitation and wind speed is stronger. In supplementary information, the regional and global climate models are compared to an ensemble of high-resolution CMIP6 and against ERA5 reanalysis data, to assess their ability in reproducing the seasonal cycle (Fig. S8) and spatial distribution (Fig. S9) of intense Mediterranean cyclones during the historical period (1985–2014).

**Figure 1:**
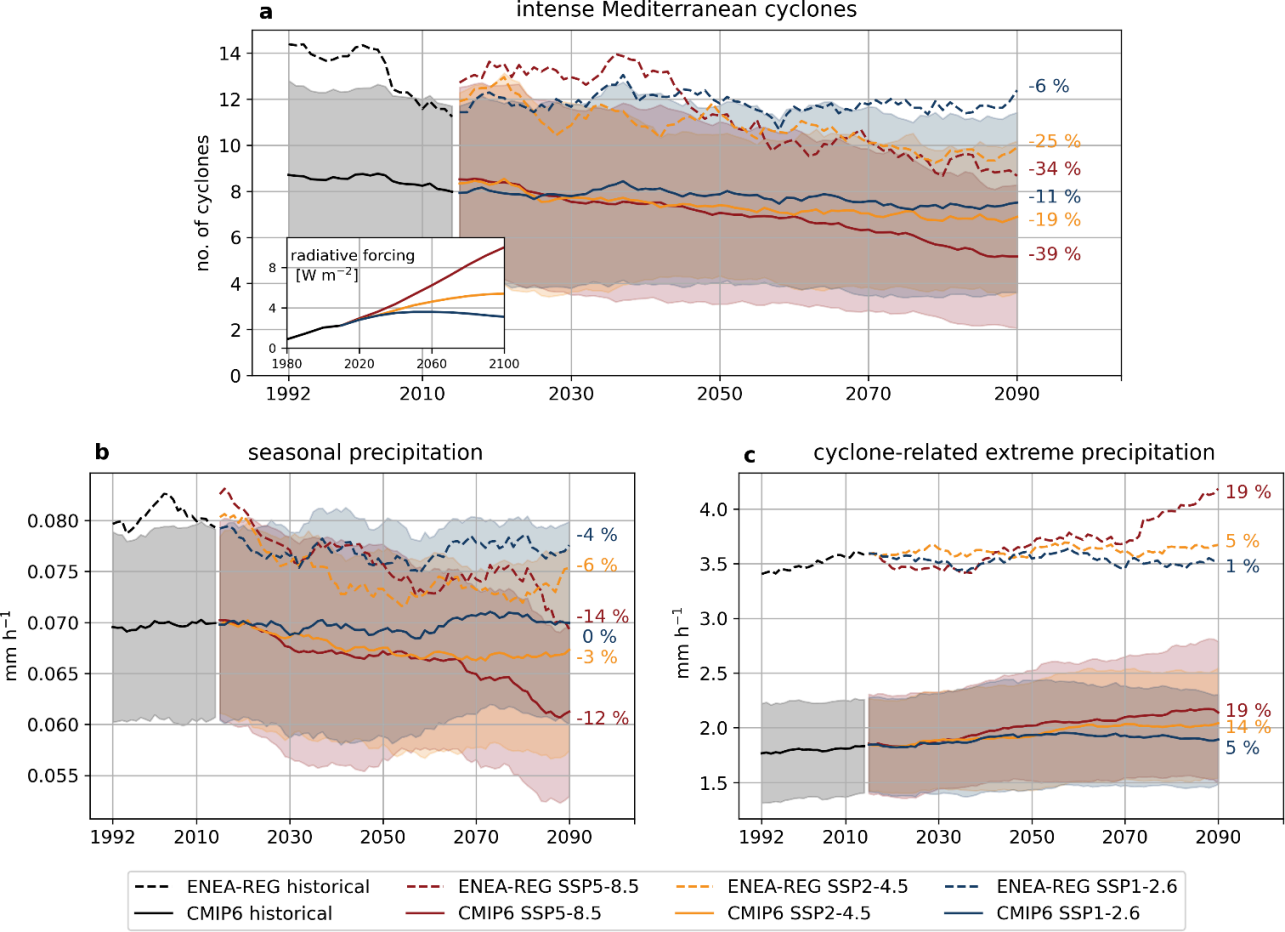
Temporal evolution of intense Mediterranean cyclones and associated extreme precipitation. Time series for (a) the number of intense Mediterranean cyclones, (b) seasonal Mediterranean precipitation (averaged over the Mediterranean domain outlined by dashed lines in Figure S1) and (c) the extreme (99^th^ percentile) precipitation associated with intense Mediterranean cyclones (averaged over the number of cyclones each year). All time series are smoothed using a 20-year moving average. Results are shown for the ONDJFM cold season in ENEA-REG (dashed lines) and the CMIP6 ensemble (solid lines), covering the historical period (black lines) and future scenarios SSP 5–8.5 (red), SSP2–4.5 (orange) and SSP1–2.6 (blue). The colour bands indicate the standard deviation for the CMIP6 ensemble. The percentages at the end of the time series show the changes of the end of the century compared to the historical mean. Panel (a) includes an inset showing the evolution of anthropogenic radiative forcing under different SSP scenarios.

During the extended cold season, both ENEA-REG and the ensemble of CMIP6 simulate a significant decrease in the number of intense Mediterranean cyclones (34–39% under SSP5–8.5; Fig. 1a) and in seasonal precipitation (12–14% under SSP5–8.5; Fig. 1b) with increasing greenhouse gas (GHG) emissions across the three analysed SSP scenarios. In clear contrast, extreme (99^th^ percentile) precipitation associated with these cyclones (Fig. 1c) is projected to increase by up to ~ 19% by the end of the century under the high-emission scenario (SSP5–8.5). This intensification aligns with radiative forcing trends under the various emission scenarios^14^ (inset Fig. 1a), as increased energy in the atmosphere fuels these cyclonic events. Notably, ENEA-REG simulates more intense cyclones and stronger extreme precipitation than the CMIP6 ensemble, benefiting from its finer resolution^16^.

Despite the large spread in future projections, reflected by the standard deviation among CMIP6 models (shaded areas in Fig. 1), our analysis highlights the important role of Mediterranean cyclones on the extreme precipitation trends and reinforces the climate paradox for the Mediterranean basin^9^: if GHG emissions continue to rise, the region will experience lower seasonal precipitation due to reduced cyclonic activity, while even more extreme precipitation during intense cyclones. The combination of increased aridity and amplified precipitation extremes is expected to heighten the risk of inland flooding from these storms, posing substantial challenges for the densely populated areas of the Mediterranean^17^. These findings motivate further investigation into the regional patterns of cyclone-related precipitation changes and their underlying physical drivers.

**Figure 2:**
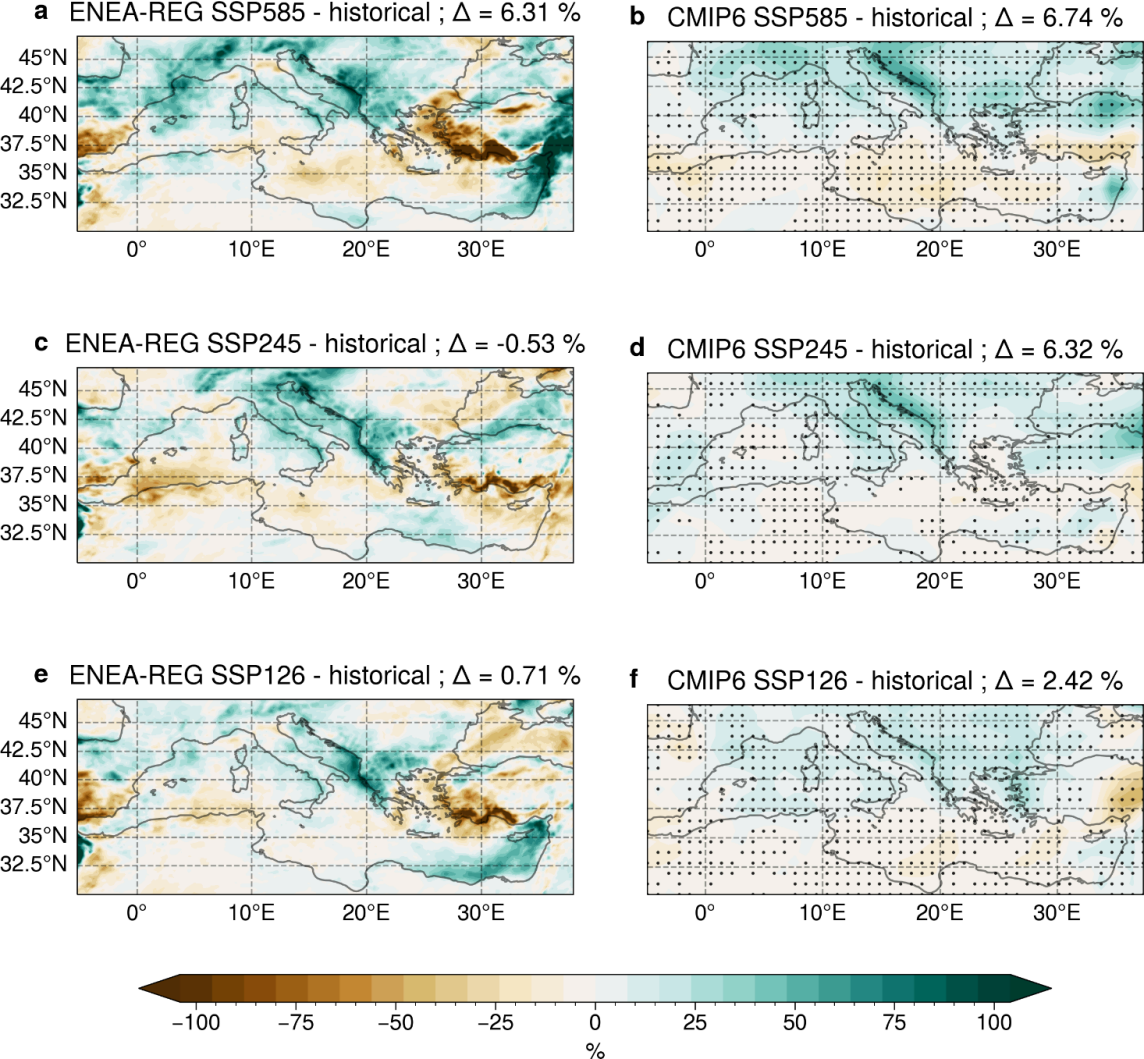
Cyclone-related precipitation changes. Maps of the differences in precipitation associated with intense Mediterranean cyclones between the far future (2071–2100) and the historical period (1985–2014), for SSP 5–8.5, 2–4.5 and 1–2.6 scenarios. Results are shown for the ONDJFM cold season in ENEA-REG (panels a, c, e) and the CMIP6 ensemble (panels b, d, f). The differences are normalized by the historical mean value of cyclone-related precipitation. Black points on the CMIP6 maps indicate areas where at least four out of six models (i.e., more than 50%) agree on the sign of the difference. Δ values represent the domain average of the differences.

Figure 2 shows the regional patterns of the cyclone-related precipitation changes between the far-future (2071–2100) and the historical period (1985–2014). Higher levels of radiative forcing lead to intensification of the precipitation responses both in the regional and global models, with the former showing more pronounced changes. In all scenarios, we observe a patchy response, with precipitation increasing in northern, western and central Mediterranean regions, as well as along the Levantine coast, while decreasing in southern regions, especially over the Ionian and Aegean Seas and the Turkish coast. These changes either counterbalance (in central areas) or amplify (in southern regions) the dry effects caused by the overall reduction in cyclone frequency. More specifically, while the frequency of intense storms is expected to decrease significantly under the high emission scenario (SSP5–8.5) in the central Mediterranean (Fig. S2a and b), reducing seasonal precipitation (Fig. S3a and b), the models project a marked increase in cyclone-related precipitation (Fig. 2a, b), particularly over the Italian Peninsula and Balkan regions, where storms typically produce heavy rainfall (Fig. S4). This intensification is driven by the interaction between the cyclones and the complex orography of the basin, leading to intense precipitation over coastal areas, a feature captured only by the AORCM (ENEA-REG vs. CMIP6 in Fig. 2 and S4).

**Figure 3:**
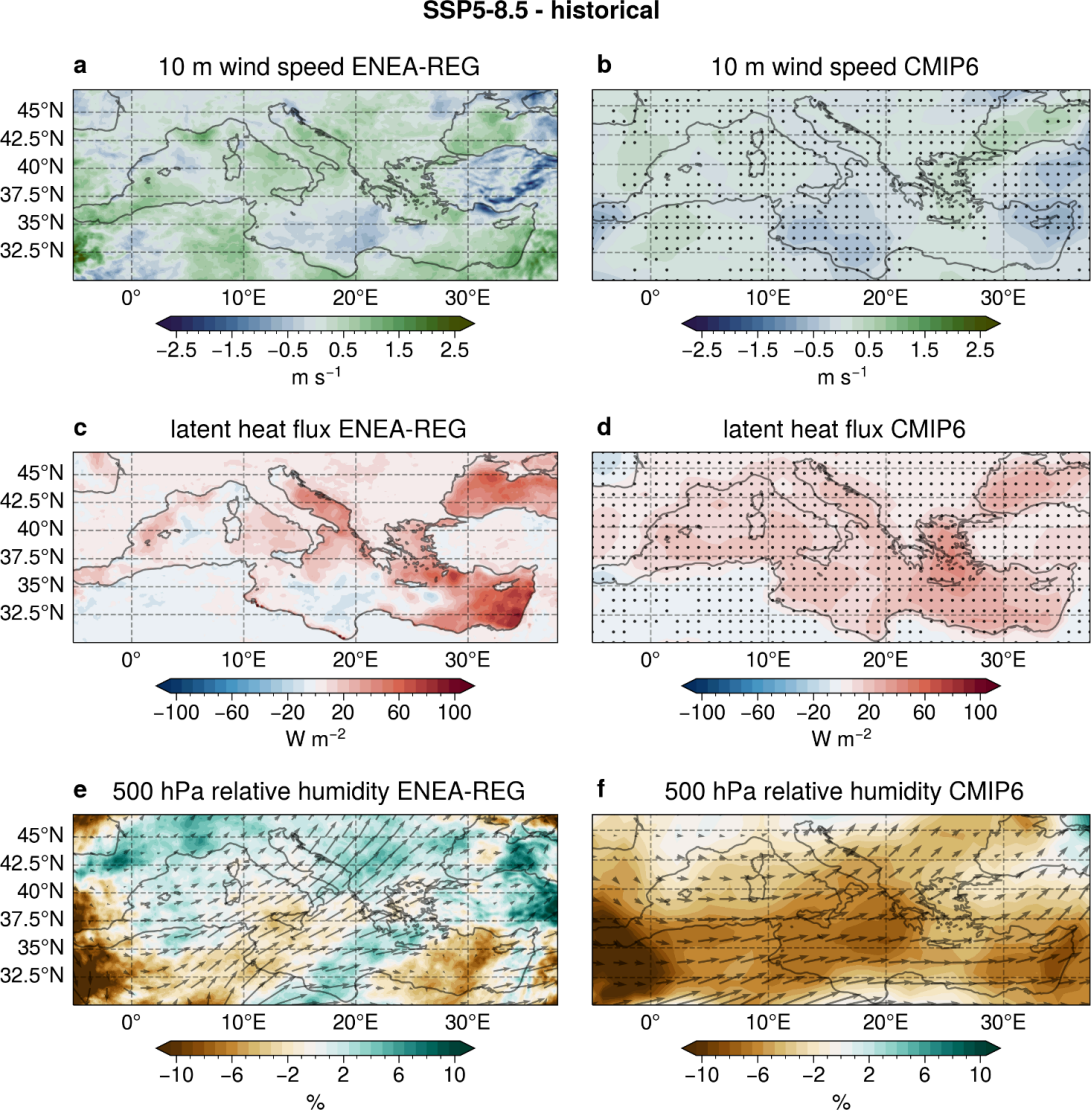
Cyclone-related atmospheric field changes for SSP 5–8.5. Maps of the differences in 10 m wind speed (a, b), latent heat flux (c, d) and moisture transport (black arrows) and relative humidity (shaded areas) at 500 hPa (e, f) associated with intense Mediterranean cyclones between the far future (2071–2100) and the historical period (1985–2014) for SSP 5–8.5 scenario. Results are shown for the ONDJFM cold season in ENEA-REG (panels a, c, e) and the CMIP6 ensemble (panels b, d, f). Black points on the CMIP6 maps indicate areas where at least four out of six models (i.e., more than 50%) agree on the sign of the difference. The moisture transport is computed as the product of specific humidity and the horizontal wind speed vector, with arrows indicating the direction of the difference. In the CMIP6 map (f), arrows are shown only for areas where at least four out of six models agree on the sign of the difference.

The projected increase in cyclone-related precipitation with climate change results from a combination of moisture convection and advection processes. In the ENEA-REG simulations, Mediterranean cyclones produce stronger 10 m wind speeds across most of the Sea under different emission scenarios (Fig. 3a, S5a, S6a). This, along with rising sea surface temperatures (SST, Fig. S7), fosters latent heat flux in the same areas (Fig. 3c, S5c, S6c), enhancing the vertical heat and moisture exchange from the ocean to the atmosphere during these storms. Additionally, at the mid-troposphere (500 hPa), increased moisture transport from the southwest (black arrows in Fig. 3e) provides further precipitable water to cyclones over the central Mediterranean. As a result, convection and advection processes together lead to higher relative humidity over the Italian Peninsula and the Balkan, captured only by the AORCM (shaded areas in Fig. 3e, S5e, S6e), which explains the increase in cyclone-related precipitation. In contrast, the southern Mediterranean is projected to experience pronounced drying due to a northward shift in storm tracks and a weakening of cyclone baroclinic forcing^9^. These changes lead to higher atmospheric stability and inhibition of convection, reducing 10 m wind speed and latent heat flux over the Ionian Sea (visible only in ENEA-REG, Fig. 3a and c), which is the primary moisture source for storms in this region.

These results highlight the ability of a high-resolution AORCM to coherently simulate the interconnected dynamic and thermodynamic processes during intense Mediterranean cyclones, providing new insights into how moisture is supplied during these events under different emission scenarios. This improvement stems from the model’s finer representation of wind patterns and air-sea fluxes which influence cyclone-related precipitation^18,19^. Additionally, compared to the coarse CMIP6 models, AORCMs enhance the representation of Mediterranean Sea circulation^20^ by resolving mass transport and energy flux exchanges through the Strait of Gibraltar^13^. Future changes in ocean circulation patterns (cyclonic and anticyclonic) modulate air-sea fluxes, affecting cyclone-related precipitation response^21^. While a multi-model ensemble helps identify robust precipitation signals under climate change (Fig. 2b, d, f), CMIP6 models struggle to capture the interaction between the Mediterranean Sea circulation and the local energy exchanges with the atmosphere, specifically the relation between changes in 10 m wind speed (Fig. 3a vs. 3b) and latent heat flux (Fig. 3c vs. 3d). In addition, they fail to accurately simulate the dynamics of moisture transport and the resulting increase in relative humidity (Fig. 3e vs. Figure 3f) over areas with complex orography.

These findings provide novel insights into the key role of cyclones on both the changing mean climate and extremes in the Mediterranean, offering valuable information for regional climate impact assessments and adaptation strategies. The analysis underscores the potential risk of more damaging inland flooding in a warming climate, particularly in southern Spain and France (including their islands), the Italian Peninsula, the Balkan region and the Levantine Coast, posing severe issues for such densely populated areas. Besides, our results highlight the importance of combing different models within coordinated frameworks to disentangle the influences of large-scale forcings and regional climate processes on the future Mediterranean climate under varying radiative forcing levels. This approach is crucial for improving climate modelling and climate projections reliability.

## Methods

### Models description

One high-resolution atmosphere-ocean coupled regional climate model (ENEA-REG^13^) and six CMIP6 global climate models (Table 1) are used in this study. Four different simulation periods are analysed, i.e. one historical (1982–2014) and three future scenarios (2015–2100): SSP5–8.5, SSP2–4.5 and SSP1–2.6.

ENEA-REG^13^ simulates various components of the Earth system. The atmosphere is represented using the Weather Research and Forecasting model (WRF, version 4.2.2), the land processes are simulated using the Noah-MP scheme embedded within WRF, the Ocean circulation is reproduced using the Massachusetts Institute of Technology General Circulation Model (MITgcm, version Z67^22^), and freshwater fluxes are modelled using the Hydrological Discharge model (HD, version 1.0.2^23^). The model domain covers the Med-CORDEX region (Fig. S1) with a horizontal resolution of 12 km for the atmospheric component and approximately 10 km (1/12°) for the ocean component. In this study, ENEA-REG is initialised and forced by the MPI-ESM1–2-HR global model. The first two years of the historical simulation (1980–1982) are used as a spin-up period and thus the analysis is performed over the 1982–2100 timeframe.

The six CMIP6 global climate models were selected based on specific criteria. These criteria include a horizontal resolution finer than 100 km, comparable to that of the parent global model of ENEA-REG (MPI-ESM1–2.HR) and the availability of 6-hourly Sea Level Pressure (SLP) data required for the cyclone tracking algorithm. In addition, in the supplementary information, seven CMIP6-HR high-resolution global climate models are analysed for the historical experiment (Table 1), as the SSP scenarios are not available for these models. These high-resolution global models were chosen based on the availability of 6-hourly SLP data to meet the requirements of the tracking method.

### Cyclone tracking algorithm

To identify Mediterranean cyclones, a cyclone tracking algorithm is applied to ENEA-REG, CMIP6 and CMIP6-HR simulations, as well as to ERA5 reanalysis, over the Med-CORDEX domain (Fig. S1). The tracking method is the same of Flaounas et al. (2023)^24^ called “CycloTRACK”, adapted from Flaounas et al. (2014)^25^ and uses Mean Sea Level Pressure (MSLP), at 6 hourly intervals, to detect cyclone centres. Initially, a Gaussian filter with a 150 km kernel and a sigma value of 2 is applied to smooth the MSLP field. Cyclone centres are then identified as grid points with lower MSLP than their eight surrounding neighbours. Starting from the cyclone centre with deepest MSLP, the algorithm constructs potential tracks by connecting centres across consecutive time steps within a 250 km radius. From these candidates, the track with the smallest average MSLP difference is selected.

The MSLP fields of ENEA-REG, CMIP6 and CMIP6-HR are interpolated onto ERA5’s regular grid (0.25°) before applying the tracking algorithm, to assure comparability between the models and the reanalysis^26^. Cyclones over areas with altitudes above 800 m are filtered out, to exclude cyclone artefacts that form over mountains due to pressure field extrapolation to sea level^27^. Only cyclones with a minimum SLP below 1000 hPa and located within the area outlined by solid lines in figure S1^6^ are classified as “intense Mediterranean cyclones” and included in this study. This allows to remove small cyclonic features that typically have limited influence on climate dynamics and extremes of the Mediterranean area^7^.

The same methodology has been already applied in the hindcast (ERA5 driven) simulation of ENEA-REG, which highlighted how the model accurately represents the cyclone track statistics, i.e. intensity, lifetime and speed, as well as season cycle and spatial distribution^19^.

### Cyclone-related fields

To investigate the physical mechanism driving changes in cyclone-related precipitation, several fields have been analysed, including precipitation, 10 m wind speed, SST, latent heat flux, relative humidity at 500 hPa and mid-level moisture transport (specific humidity multiply be the wind speed vector at 500 hPa). The analysis is performed for both the regional and the global CMIP6 models at their original spatial resolutions. These fields are computed during the mature stage of each cyclone, defined as the timestep of the minimum SLP plus the two steps before and after it, totalling five time steps. The analysis is restricted to the area of influence of the cyclones, i.e. a circular disk with a 1000 km radius^6^ around each SLP tracking points of the mature stage. To note that the models’ output frequency is 6 hours, thus the mature stage spans from 12 hours before to 12 hours after the time of the minimum SLP. For each grid point within the Mediterranean area (outlined by dashed lines in Fig. S1), the fields are averaged over all occurrences when a cyclone passes through that cell. These averaged fields are referred to as “cyclone-related fields”. The precipitation differences are normalised by the mean historical value over the domain and expressed in percentage, Eq. (1):

1. $$\Delta =\frac{100\;(\mathrm{future}\;-\;\mathrm{historical})}{\mathrm{historical}\;\mathrm{mean}}\%$$

The extreme precipitation associated with intense Mediterranean cyclones (Fig. 1c) is computed for each cyclone as the 99^th^ percentile of precipitation during its mature stage and within its area of influence (i.e. circular disk with a 1000 km radius). In the time series shown in Fig. 1c, each year’s value represents the average extreme precipitation across all cyclones that occurred in that year.

## Figures and Tables

**Table 1:** List of analysed regional and global climate models. The models include ENEA-REG, the CMIP6 and the CMIP6-HR.

Type of model	Model name	Institution	Atmospheric horizontal resolution
Regional climate model	ENEA-REG	ENEA, Italy	12 km
CMIP6	BCC-CSM2-MR	BCC, China	100 km
CMIP6	CMCC-ESM2	CMCC, Italy	100 km
CMIP6	EC-Earth3	European Consortium, Europe	80 km
CMIP6	MPI-ESM1.2-HR	MPI, Germany	80 km
CMIP6	MRI-ESM-2.0	MRI, Japan	100 km
CMIP6	NorESM2-MM	NCC, Norway	100 km
CMIP6-HR	BCC-CSM2-HR	BCC, China	40 km
CMIP6-HR	CMCC-CM2-VHR4	CMCC, Italy	20 km
CMIP6-HR	CNRM-CM6.1-HR	CNRM, France	50 km
CMIP6-HR	EC-Earth3P-HR	European Consortium, Europe	40 km
CMIP6-HR	ECMWF-IFS-HR	ECMWF, Europe	30 km
CMIP6-HR	GFDL-CM4C192	NOAA-GFDL, USA	50 km
CMIP6-HR	MPI-ESM1.2-XR	MPI, Germany	40 km

## Data Availability

Enquiries about data availability should be directed to the authors.

## References

[1] Pozzer G. Assessing Hydrogeological Vulnerability Within Northern Apennines: An Integrated Spatial Analysis in Emilia-Romagna Region (Italy). in 7–33 (2024). doi:10.1007/978-3-031-65463-3_2.

[2] Piccolroaz S. Devastating Spanish floods expose an urgent need for more flood-risk professionals. Nature 635, 290–290 (2024). 10.1038/d41586-024-03703-9 39533079

[3] Giorgi F. Climate change hot-spots. Geophys Res Lett 33, (2006).

[4] Tuel A. & Eltahir E. A. B. Why Is the Mediterranean a Climate Change Hot Spot? J Clim 33, 5829–5843 (2020).

[5] Flaounas E. Mediterranean cyclones: current knowledge and open questions on dynamics, prediction, climatology and impacts. Weather and Climate Dynamics vol. 3 173–208 Preprint at 10.5194/wcd-3-173-2022 (2022).

[6] Reale M. Future projections of Mediterranean cyclone characteristics using the Med-CORDEX ensemble of coupled regional climate system models. Clim Dyn 58, 2501–2524 (2022).

[7] Flaounas E., Gray S. L. & Teubler F. A process-based anatomy of Mediterranean cyclones: from baroclinic lows to tropical-like systems. Weather and Climate Dynamics 2, 255–279 (2021).

[8] Allan R. P. Advances in understanding large-scale responses of the water cycle to climate change. Ann N Y Acad Sci 1472, 49–75 (2020). 32246848 10.1111/nyas.14337

[9] Zappa G., Hawcroft M. K., Shaffrey L., Black E. & Brayshaw D. J. Extratropical cyclones and the projected decline of winter Mediterranean precipitation in the CMIP5 models. Clim Dyn 45, 1727–1738 (2015).

[10] Seager R. Causes of Increasing Aridification of the Mediterranean Region in Response to Rising Greenhouse Gases*. J Clim 27, 4655–4676 (2014).

[11] Insua-Costa D., Senande-Rivera M., Llasat M. C. & Miguez-Macho G. A global perspective on western Mediterranean precipitation extremes. NPJ Clim Atmos Sci 5, 9 (2022).

[12] Xie S.-P. Towards predictive understanding of regional climate change. Nat Clim Chang 5, 921–930 (2015).

[13] Anav A. Dynamical downscaling of CMIP6 scenarios with ENEA-REG: an impact-oriented application for the Med-CORDEX region. Clim Dyn (2024) doi:10.1007/s00382-023-07064-3.

[14] O’Neill B. C. The Scenario Model Intercomparison Project (ScenarioMIP) for CMIP6. Geosci Model Dev 9, 3461–3482 (2016). 10.5194/gmd-9-4521-2016 PMC5911933 29697697

[15] Ruti P. M. Med-CORDEX Initiative for Mediterranean Climate Studies. Bull Am Meteorol Soc 97, 1187–1208 (2016).

[16] Flaounas E., Drobinski P. & Bastin S. Dynamical downscaling of IPSL-CM5 CMIP5 historical simulations over the Mediterranean: benefits on the representation of regional surface winds and cyclogenesis. Clim Dyn 40, 2497–2513 (2013).

[17] Cramer W. Climate change and interconnected risks to sustainable development in the Mediterranean. Nat Clim Chang 8, 972–980 (2018).

[18] Berthou S. Influence of submonthly air–sea coupling on heavy precipitation events in the Western Mediterranean basin. Quarterly Journal of the Royal Meteorological Society 142, 453–471 (2016).

[19] Chericoni M., Fosser G., Flaounas E., Sannino G. & Anav A. Extreme Mediterranean cyclones and associated variables in an atmosphere-only vs an ocean-coupled regional model. Preprint at 10.5194/egusphere-2024-2829 (2024).

[20] Parras-Berrocal I. M. Response of the Mediterranean Sea Surface Circulation at Various Global Warming Levels: A Multi-Model Approach. Geophys Res Lett 51, (2024).

[21] Jangir B., Mishra A. K. & Strobach E. Effects of Mesoscale Eddies on the Intensity of Cyclones in the Mediterranean Sea. Journal of Geophysical Research: Atmospheres 128, (2023).

[22] Marshall J., Adcroft A., Hill C., Perelman L. & Heisey C. A finite-volume, incompressible Navier Stokes model for studies of the ocean on parallel computers. J Geophys Res Oceans 102, 5753–5766 (1997).

[23] Hagemann S. & Gates L. D. Validation of the hydrological cycle of ECMWF and NCEP reanalyses using the MPI hydrological discharge model. Journal of Geophysical Research: Atmospheres 106, 1503–1510 (2001).

[24] Flaounas E. A composite approach to produce reference datasets for extratropical cyclone tracks: application to Mediterranean cyclones. Weather and Climate Dynamics 4, 639–661 (2023).

[25] Flaounas E., Kotroni V., Lagouvardos K. & Flaounas I. CycloTRACK (v1.0) – tracking winter extratropical cyclones based on relative vorticity: sensitivity to data filtering and other relevant parameters. Geosci Model Dev 7, 1841–1853 (2014).

[26] Kouroutzoglou J., Flocas H. A., Keay K., Simmonds I. & Hatzaki M. Climatological aspects of explosive cyclones in the Mediterranean. International Journal of Climatology 31, 1785–1802 (2011).

[27] Neu U. IMILAST: A Community Effort to Intercompare Extratropical Cyclone Detection and Tracking Algorithms. Bull Am Meteorol Soc 94, 529–547 (2013).

